# zDB: bacterial comparative genomics made easy

**DOI:** 10.1128/msystems.00473-24

**Published:** 2024-06-28

**Authors:** Bastian Marquis, Trestan Pillonel, Alessia Carrara, Claire Bertelli

**Affiliations:** 1 Lausanne University Hospital and University of Lausanne, Institute of Microbiology, Lausanne, Switzerland; Pacific Northwest National Laboratory, Richland, Washington, USA

**Keywords:** comparative genomics, microbial genomics, genome visualization

## Abstract

The analysis and comparison of genomes rely on diﬀerent tools for tasks such as annotation, orthology prediction, and phylogenetic inference. Most tools are specialized for a single task, and additional eﬀorts are necessary to integrate and visualize the results. To ﬁll this gap, we developed zDB, an application integrating a Nextﬂow analysis pipeline and a Python visualization platform built on the Django framework. The application is available on GitHub (https://github.com/metagenlab/zDB) and from the bioconda channel. Starting from annotated Genbank ﬁles, zDB identiﬁes orthologs and infers a phylogeny for each orthogroup. A species phylogeny is also constructed from shared single-copy orthologs. The results can be enriched with Pfam protein domain prediction, Cluster of Orthologs Genes and Kyoto Encyclopedia of Genes and Genomes annotations, and Swissprot homologs. The web application allows searching for speciﬁc genes or annotations, running Blast queries, and comparing genomic regions and whole genomes. The metabolic capacities of organisms can be compared at either the module or pathway levels. Finally, users can run queries to examine the conservation of speciﬁc genes or annotations across a chosen subset of genomes and display the results as a list of genes, Venn diagram, or heatmaps. Those features make zDB useful for both bioinformaticians and researchers more accustomed to laboratory research.

**IMPORTANCE:**

Genome comparison and analysis rely on many independent tools, leaving to scientists the burden to integrate and visualize their results for interpretation. To alleviate this burden, we have built zDB, a comparative genomics tool that includes both an analysis pipeline and a visualization platform. The analysis pipeline automates gene annotation, orthology prediction, and phylogenetic inference, while the visualization platform allows scientists to easily explore the results in a web browser. Among other features, the interface allows users to visually compare whole genomes and targeted regions, assess the conservation of genes or metabolic pathways, perform Blast searches, or look for speciﬁc annotations. Altogether, this tool will be useful for a broad range of applications in comparative studies between two and hundred genomes. Furthermore, it is designed to allow sharing of data sets easily at a local or international scale, thereby supporting exploratory analyses for non-bioinformaticians on the genome of their favorite organisms.

## INTRODUCTION

Since the publication of the ﬁrst complete genome in 1995 (1), the number of available sequences has kept on growing, with now 450,000 diﬀerent species available in the Genbank database (2). As recent sequencing technologies make it possible to sequence an organism in a matter of hours at a cost aﬀordable even for small research laboratories, this trend is unlikely to abate in the future. These technological improvements transferred the burden from sequencing to the actual analysis of the sequences. While a plethora of diﬀerent tools already exist for this purpose, they are often specialized for speciﬁc tasks such as gene calling, orthology prediction, or phylogenetic inference. Moreover, these tools are often standalone programs that do not readily integrate each other’s results. As the results are often not produced in a format that easily allows their exploration, additional visualization eﬀorts are also necessary.

The need for tools designed to aggregate results from diﬀerent sources has been illustrated by the success of programs like Prokka (3), which merges the results of diﬀerent annotation tools in ﬁles ready for submission and visualization in genome browsers. The idea is further extended by pipelines like Bactopia (4), TORMES (5), and ASA3P (6) that automate all steps from reads quality control to antibiotic resistance gene prediction and generate simple HTML reports, allowing the visualization of the main results. As these pipelines were developed with a focus on clinical microbiology, they are limited in terms of comparative genomics analysis. In contrast, websites dedicated to the comparative genomics of speciﬁc groups of organisms (7, 8) have been developed and implemented powerful interfaces allowing users to make custom queries and generate complex plots. However, these websites do not allow users to analyze their own sets of genomes. Some web-based comparative genomics platforms, like EDGAR (9), Pathogenwatch (10), PhyloCloud (11), KBase (12), CoGe (13), or MicroScope (14) implement similar interfaces while allowing users to upload their own data set. Some of those platforms are, however, closed-source, and as the analysis is performed on the platform’s respective servers, users are required to register and upload their data set. The ideal comparative genomics platform would, therefore, be open source, could run on any infrastructure, be as ﬂexible and scalable as Bactopia, and, similar to MicroScope and EDGAR, oﬀer an extensive interface to visualize the results. The developers of anvi’o (15), OpenGenomeBrowser (16), and bioBakery (17), all of which implement an analysis pipeline and a visualization platform, demonstrate the feasibility of such an approach.

Using a similar design, we developed zDB, an open-source comparative genomics analysis pipeline and visualization platform. The analysis pipeline performs functional annotations, orthology, and phylogenetic inference, while the visualization platform oﬀers an interactive modern web-based interface to explore the results. Altogether, the ease of installing and executing the tool and the ability to easily visualize the results will beneﬁt both bioinformaticians and researchers more accustomed to lab work.

## RESULTS

The visualization platform can be started by a single command as soon as the analyses are complete. The command starts a web server that will make the results available via a web browser, either only locally or also possibly extended to the whole Internet depending on the setup. The platform can also be used to visualize archived results imported from another computer.

### Visualization toolkit

The visualization platforms implement a set of plots and queries to explore the results of orthology prediction and phylogeny inference. In addition, zDB comes with several features of more general interest like the possibility to run Blast queries, to search for speciﬁc annotation or gene using a search bar and to draw interactive Circos plots or genomic regions.

**Fig 1:**
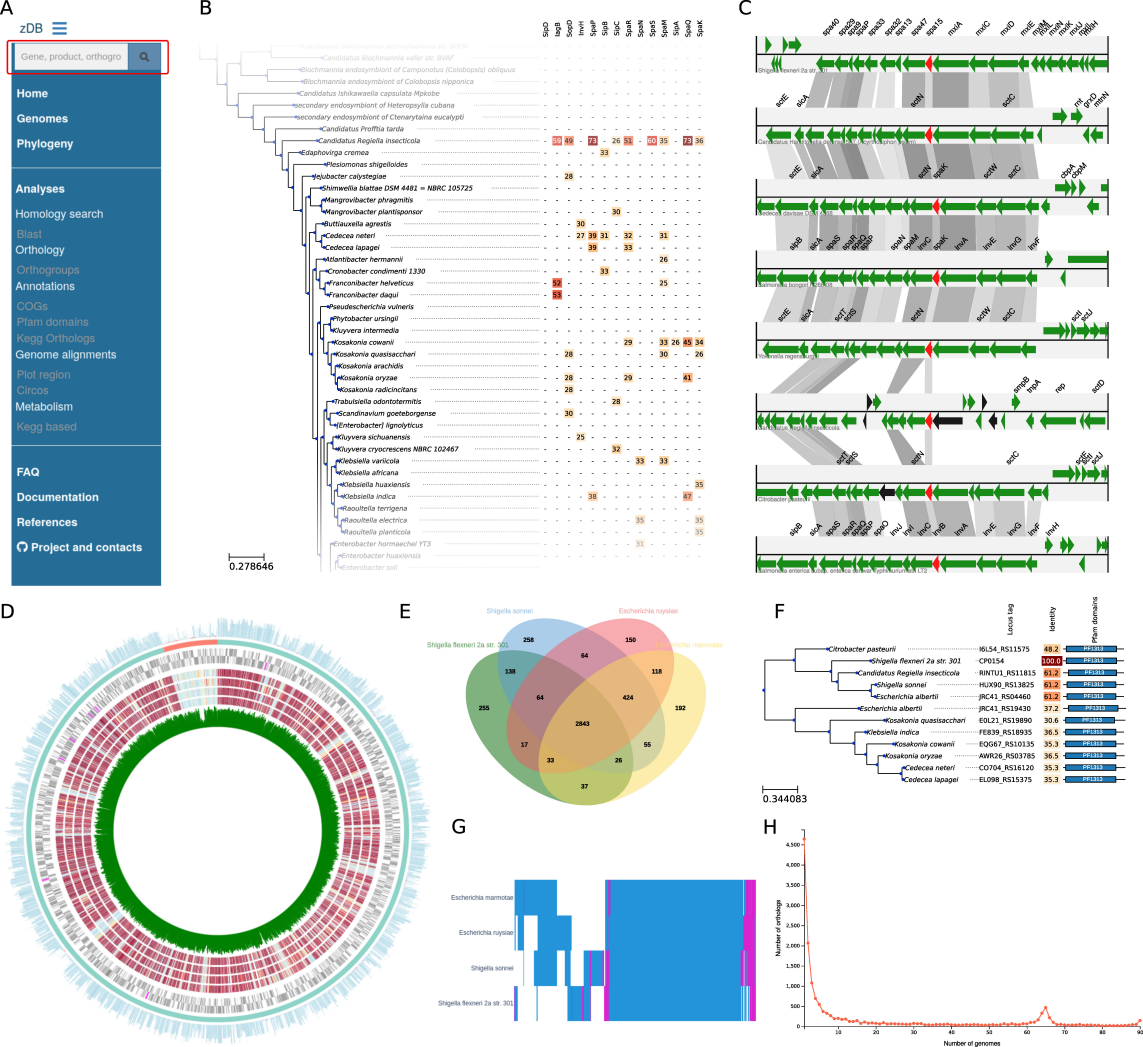
(**A**) zDB side panel listing all available analyses. The search bar (red box) allows users to look for a gene or annotation of interest. (**B**) Blast search result. The whole data set was searched for several proteins of the type III secretion system of *Salmonella* Typhimurium. Blast hits are displayed as a heatmap of identities. (**C**) zDB can draw a comparison of the genomic region of a speciﬁed gene of interest and its orthologs (in red) in several genomes. Black arrows represent pseudogenes. Gray bars link orthologous genes, and their shades reﬂect amino-acid identity. (**D**) Visualization of protein conservation of a reference genome (here *Shigella ﬂexneri*) compared to four selected genomes (here from *Escherichia* genus). The inner circle represents the GC content of each open reading frame (ORF) in the reference genome. The next four circles represent the absence (in blue) or presence (in red) of homologs of proteins from the reference genome in the selected genomes, with a color scale representing protein identity. The next two circles represent the localization of the ORFs on the forward and reverse strands of the reference genome. The two outer circles represent the contigs in the reference assembly and a histogram of the number of homologs to each protein of the reference genome in the selected genomes. (**E**) Venn diagram illustrating the distribution of the orthologous groups in four genomes. (**F**) Gene phylogeny. The identity column shows the identity relative to the CP0154 locus, as it was accessed through the page dedicated to this locus. The rightmost columns display the domain architecture. (**G**) Heatmap of gene conservation in the four genomes as in (**E**); pink bars represent genes present in multiple copies in a genome, and blue bars represent single copy genes. (**H**) Distribution of the orthologous groups in the function of the number of genomes where they occur.

A sidebar is present on all pages of the web application (Fig. 1A) and allows quick access to all available analyses. The content of the “Annotations” tab varies in function of which optional analyses were performed. Similarly, the “Metabolism” tab will only be present when the genomes were annotated with Kyoto Encyclopedia of Genes and Genomes (KEGG) orthologs. Summaries of the main characteristics of the genomes of interest, including the results from CheckM, can be visualized either as lists or directly annotated in the species phylogeny (Fig. S1) via the “Genomes” and “Phylogeny” tabs, respectively. Finally, the web interface also includes a search bar (Fig. 1A) that allows users to look for genes, gene products, bacteria, or speciﬁc annotations based on their names. The search bar accepts wildcards and logical operators to combine diﬀerent search terms.

The “Orthology” tab links to pages allowing to explore gene conservation in the data set. In particular, users can visualize gene conservation across a chosen set of genomes as either heatmaps (Fig. 1G), Venn diagrams (Fig. 1E), or lists. zDB can also draw the commonly used core and pan-genome plots as well as a plot of the number of orthogroups in function of the number of genomes where they occur (Fig. 1H). The latter plot allows for a quick assessment of the number of singletons, the size of the core genome, and the detection of gene groups occurring in a subset of the genomes. Finally, zDB implements an interface that allows users to search for genes conserved in a chosen set of genomes but absent in another one.

As searching for speciﬁc sequences in organisms of interest is a frequent task, the visualization platform implements an interface that allows users to run their own Blast searches on either the whole data set or on a speciﬁc genome. Several types of blast searches can be performed (blastp, blastn, and tblastn), either with a single query or with multiple queries in FASTA format. The results are displayed interactively using the BlasterJS library (18). Moreover, if the search was run on the whole data set, zDB can display the results as a heatmap of the best blast hits identity linked to the species phylogenetic tree (Fig. 1B). This allows to quickly detect patterns in the distribution of Blast hits in function of the phylogenetic distance.

**Fig 2:**
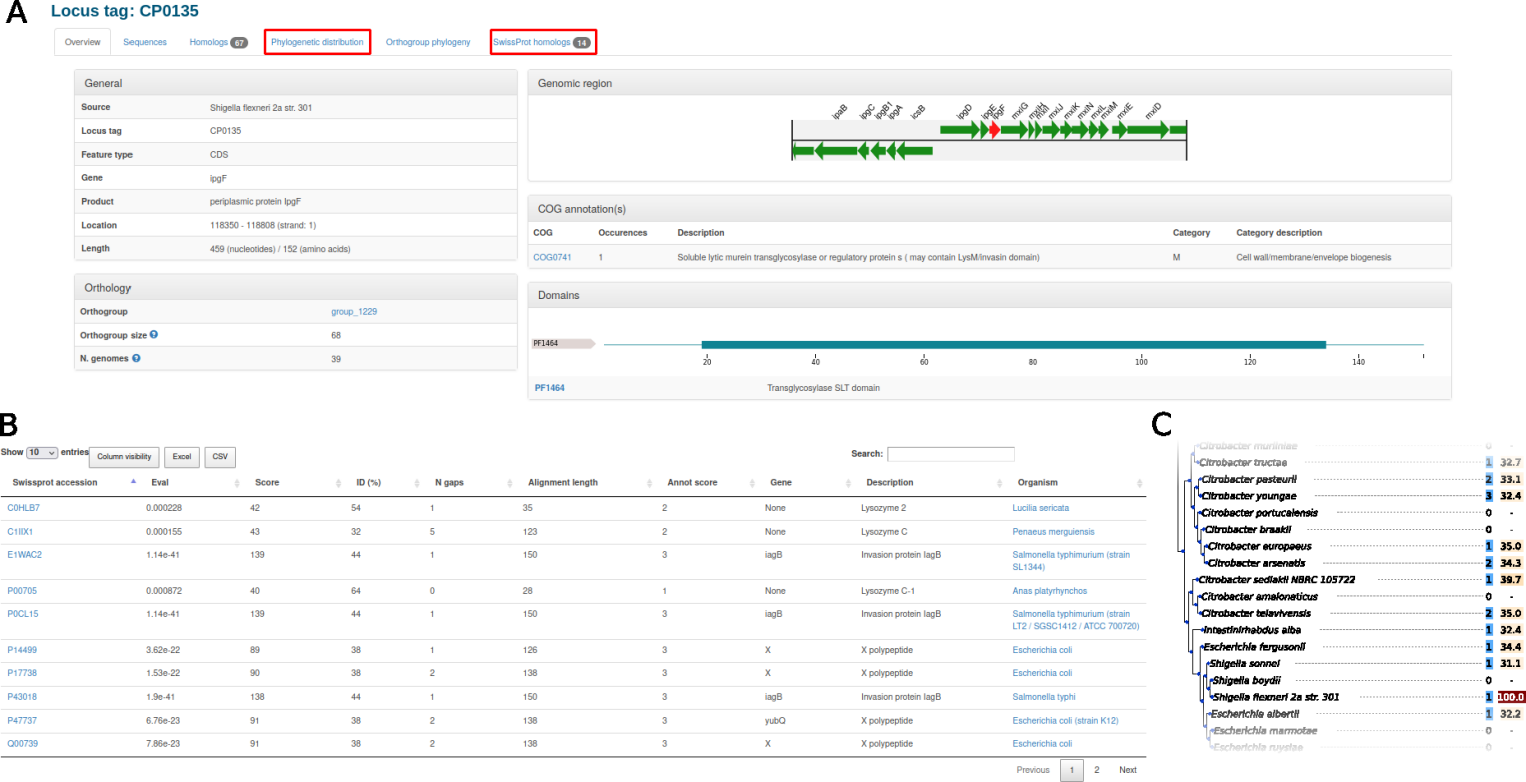
(**A**) Example of a gene summary page, with its genomic region, Pfam domains, and Cluster of Orthologs Genes (COG) annotation. The phylogenetic distribution and list of Swissprot homologs shown in (**B** and **C**) can be accessed from the highlighted tab. (**B**) The list of Swissprot homologs. (**C**) Part of the phylogenetic tree and protein conservation. The ﬁrst column shows the number of homologs of the gene in a given genome. The second column shows the amino-acid identity between the gene of interest and its closest homolog in a given genome.

Finally, zDB can draw plots to compare genomic regions sharing orthologous genes (Fig. 1C) and Circos plots to compare a set of genomes to a speciﬁed reference (Fig. 1D). The former notably allows to visually confront the orthology inference to the predicted genes order in homologous regions. The minimal setup also includes summary pages for every gene and orthogroup. The gene summary page allows easy access to nucleotide and amino-acid sequences (for protein-coding genes), displays the genomic region of the gene of interest, and provides a list of orthologous genes (Fig. 2A). The orthogroup page allows users to examine the gene phylogeny and the distribution of the orthogroup in the genomes of the data set (Fig. 1F; 2C). Both pages are enriched with the results of the optional analyses if they were performed (Fig. 2A and 1F).

### Pfam, COG, and KEGG functional analyses

**Fig 3:**
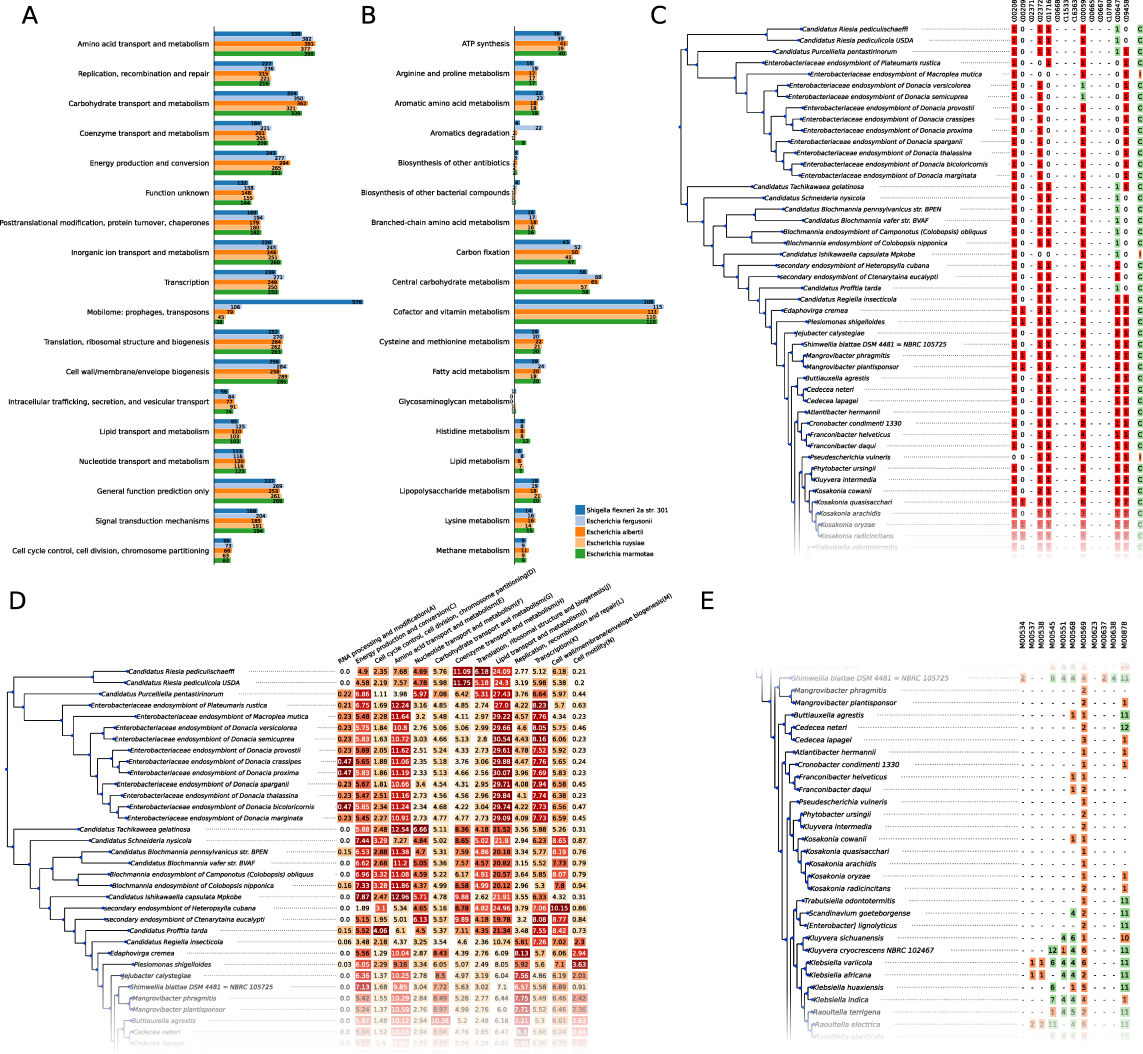
(A and **B**) Distribution of genes annotated with COG and KEGG orthologs in their functional categories for four chosen genomes. (**C**) Details of the completeness of KEGG module 83 (fatty acid biosynthesis and elongation). Red squares in the heatmap correspond to the number of genes annotated as a given KEGG ortholog. Green squares correspond to a gene without a speciﬁc KEGG annotation but in the same orthogroup as other genes having this annotation. This may indicate a shared function, and in such cases, the corresponding KEGG ortholog is considered as present when estimating the module completeness. The last column indicates module completeness, as determined by the module deﬁnition language. (**D**) Proportion of the genes in genomes assigned to the diﬀerent COG categories (some categories were removed for the sake of simplicity). (**E**) Overview of modules completeness in any given KEGG category. Green squares indicate a complete KEGG module, and orange squares indicate an incomplete module. The number of genes annotated as KEGG orthologs for a given module is indicated in each square.

The conservation of Pfam, Cluster of Orthologs Genes (COG), and KEGG annotations across genomes can be compared in a similar way to orthogroups. In particular, Venn diagrams, heatmaps, pan-, and core-genome plots can be drawn for those annotations, while an interface to search for annotations present in a set of chosen genomes but absent in another is also available. Since COG and KEGG orthologs are assigned to high-level functional categories, users can visualize the distribution of annotated genes in those categories across one or several genomes, either as bar charts (Fig. 3A and B) or as heatmaps (Fig. 3C). This allows users to quickly visualize diﬀerences of functional capabilities between organisms.

To further characterize metabolic capacities, zDB implements a parser for the KEGG module deﬁnition language, which allows to assess the completeness of a metabolic module based on the KEGG orthologs present in a genome. Module completeness can be compared at the scope of a single KEGG module (Fig. 3C) or at the scope of categories or sub-categories (Fig. 3E). The results are directly linked to the species phylogeny, making it easy to notice patterns of metabolic capacities linked to speciﬁc clades.

The Swissprot homologs are listed both on the orthogroup home page and on the gene homepage (Fig. 2B).

### Benchmarking

**Fig 4:**
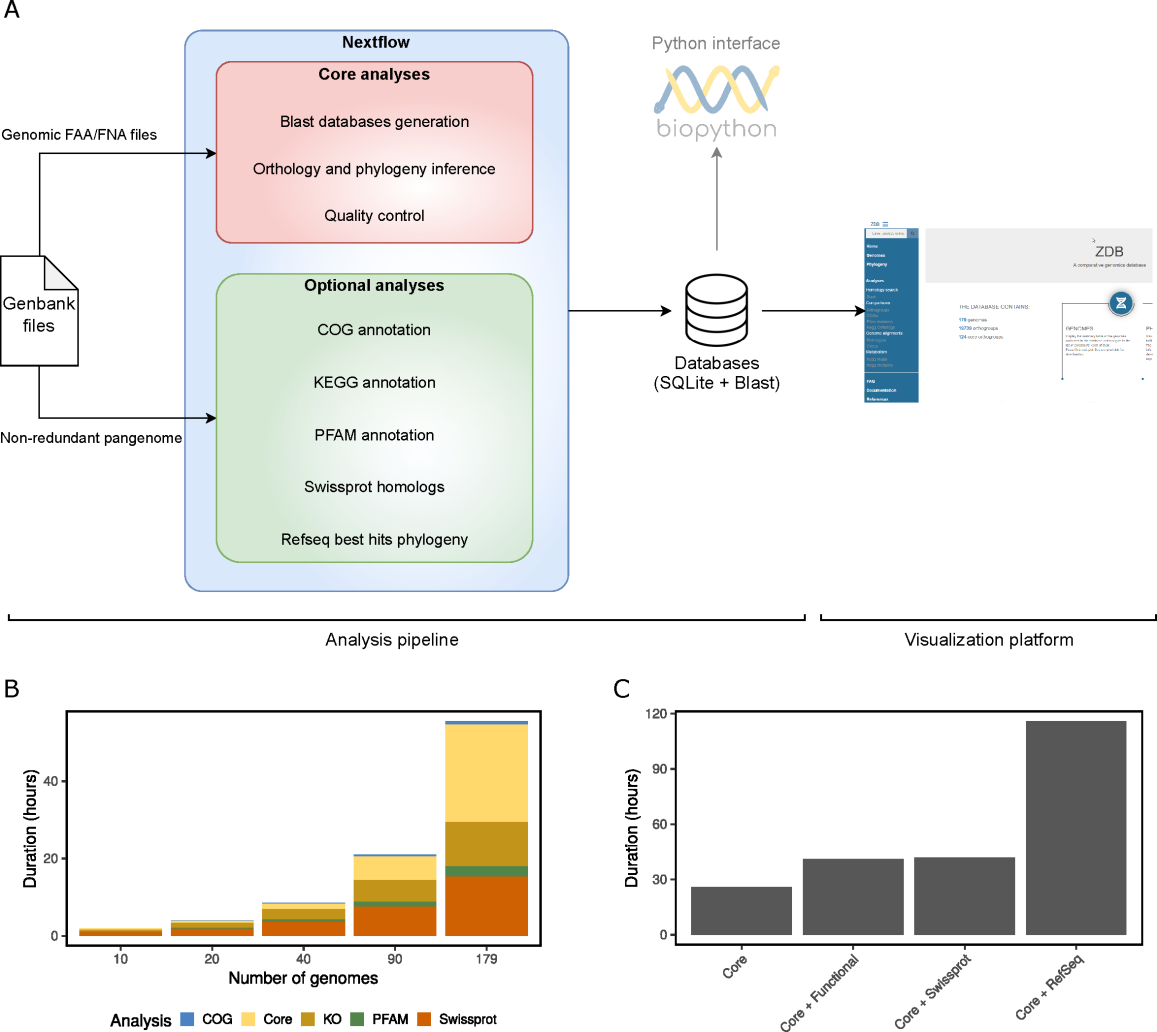
(**A**) zDB dataﬂow. The core analyses are performed for each data set, while users can choose to perform additional optional analyses. (**B**) Duration of the analysis split by type (core and optional) in total CPU time according to the number of genomes in a benchmarking. (**C**) Benchmarking of the diﬀerent analysis types with the 179 genome data set. Functional analysis includes COG and KEGG orthology annotations and Pfam domain prediction. Swissprot and RefSeq represent searching for homologs in Swissprot and Refseq, respectively.

An initial benchmark evaluated the duration of computations for data sets with an increasing number of genomes (Fig. 4B) using a conﬁguration mimicking a high-end desktop computer. Generating a database with all the optional analysis (except the RefSeq homologs search) took 1.9 h, 3.9 h, 8.6 h, 21.0 h, and 55.6 h for the data sets with 10, 20, 40, 90, and 179 genomes, respectively. The CPU (central processing unit) time spent in the optional analyses increased linearly with the number of genomes. This was, however, not the case for the core analysis. In particular, the cost of orthology prediction increased faster than the other analyses and will likely be the limiting factor for larger data sets. This is expected due to the $O(n^{2})$ complexity of the all-against-all genomes comparison performed by Orthoﬁnder (19).

The choice of optional analyses for the 179 genome data set signiﬁcantly impacts the computing time (Fig. 4C). Despite the use of Diamond instead of blastp, searching for homologs in the RefSeq database took about four times longer than the other analyses. Similarly, searching for homologs in the Swissprot database took as long as performing the KEGG, COG, and Pfam annotations together.

Finally, to conﬁrm that zDB can process 100 genomes on a desktop computer, we ran the analysis pipeline on the 90 genomes data set on a desktop machine, which took 71 h to complete.

## DISCUSSION

zDB is a comparative genomics analysis tool entirely run on the user side that includes both an analysis pipeline and a web interface to visually explore the results. It was designed to require minimal typing on the command line; only three commands from installation to visualization of the results. As shown in the benchmarks, the analysis pipeline can process data sets of 100 genomes in a matter of days on a desktop computer, making dedicated computing infrastructures unnecessary for all but the largest data sets. The possibility to easily run Blast queries, to search for speciﬁc genes, or to retrieve amino-acid and nucleotide sequences will make zDB useful for researchers more accustomed to lab work, while features such as core and pan-genome analysis and genomic regions comparisons will be useful to seasoned bioinformaticians. The ability to share the database and launch the web interface from any computer facilitates data sharing and accessibility within and across groups and institutions with minimal infrastructure. Altogether, this makes zDB a tool easy to use and install for a wide variety of applications such as genome browsing, characterization of newly sequenced genomes, or even setting up a public database for an organism of interest.

### Comparison to existing tools

Some of the features and analyses implemented in zDB overlap with those oﬀered by similar existing tools such as Anvi’o (15) and OpenGenomeBrowser (16). The pangenomics and phylogenomics workﬂows of Anvi’o are notably similar to the approach used in zDB. Single-copy core orthologs are ﬁrst identiﬁed and used to infer a phylogenetic tree that can then be visualized in the interface. OpenGenomeBrowser can infer a species’ phylogeny based on Orthoﬁnder predictions. The visualization of Blast search results or the ability to estimate KEGG module completeness is also available in OpenGenomeBrowser and Anvi’o, respectively. OpenGenomeBrowser also allows to search for genes or annotations that are statistically associated with user-speciﬁed phenotypic characteristics, while Anvi’o implements an enrichment analysis to detect functional diﬀerences between clusters of genes.

The focus of those tools is however diﬀerent. zDB was designed for comparative genomics, and as a consequence, most visualizations are articulated around orthology predictions and the inferred phylogenetic trees, allowing the recognition of phylogenetic patterns in the distribution of genes or annotations. Anvi’o being less specialized, it lacks some of the visualizations implemented in zDB but oﬀers a much broader set of analyses including the ability to handle meta-transcriptomics or meta-genomics data. While OpenGenomeBrowser was also designed for comparative genomics, it does not integrate annotations with the inferred phylogenetic tree but provides some other analyses such as dot-plot visualization of pairwise alignments of genomes.

### Limitations and future directions

Unlike other tools such as Bactopia (4), which can either use already annotated genomes or perform *de-novo* assemblies from raw reads, zDB only accepts pre-annotated genomes. While this may limit researchers not used to bioinformatics, the availability of good quality, easy-to-use annotation tools (3, 20) should not represent a signiﬁcant limitation to users. As zDB focuses on functional annotations and phylogenetic inference, we chose to leave the burden of gene prediction—and the responsibility to ensure the quality of the genomes included in the analysis—to the user. However, given the modularity of the pipeline, an optional annotation step could easily be included in future releases along *de novo* assembly and quality checks.

Large phylogenies are cumbersome to visualize as they may not entirely ﬁt on a computer screen. To alleviate this, we plan to replace the ete3 drawing engine by a custom Javascript library to draw interactive phylogenetic trees allowing the user to collapse and expand branches. As of now, the addition or removal of genomes from an existing database is not possible and requires to repeat all the analyses on the modiﬁed data set. We, therefore, plan to implement the possibility to add or remove genomes, which will allow users to incrementally improve a database without having to repeat the analyses. Finally, we will rapidly extend the set of optional analyses with additional annotations such as the prediction of antibiotic resistance genes, virulence factors, protein transmembrane domains, or signal peptides.

## MATERIALS AND METHODS

### Design and implementation

zDB is composed of two parts that can be run independently (Fig. 4A): an analysis pipeline that performs all the computationally intensive steps and stores the results in a Sqlite3 database, and a visualization platform that renders the results stored in the database in a graphical interface.

The analyses are separated in a set of core analyses focused on orthology prediction and phylogeny inference and a set of independent optional analyses, with a focus on functional annotation. To simplify the installation and make the analyses reproducible and scalable, all steps are run either within docker (21) or singularity (22) containers or in conda environments, under the control of the Nextﬂow workﬂow manager (23). Nextﬂow allows the analyses to be easily scaled from high-performance clusters to desktop machines, while containers guarantee the reproducibility and ease of installation by packaging the tools in controlled environments. By default, the analysis pipelines run the analyses in parallel, with a modiﬁable maximum of four simultaneous processes. As several of the tools used by zDB can take advantage of multi-threading, parallelism can also be applied at the analysis level by editing the conﬁguration ﬁle, although this is not enabled by default. After the completion of the analysis pipeline, zDB can export the results as a compressed archive for subsequent use. The ability to export the results was developed to facilitate sharing and to accommodate the fact that the analysis may have to be run and exported from a high-performance computing cluster, where long-term storage might not be possible due to disk space constraints.

The visualization platform was implemented as a Django website building upon the scaﬀold of ChlamDB (7). The Django server can either be instantiated on a desktop computer, for local access, or on an Internet-facing computer, if the website is to be made public (internally within a network or externally). The results are rendered as lists, annotated phylogenetic trees, and interactive plots. The phylogenetic trees are drawn as static images with the ete3 toolkit (24), while the interactive plots are generated by a collection of home-made scripts based on the d3.js framework and several libraries such as jvenn.js (25), Circos.js (https://github.com/nicgirault/circosJS), BlasterJS (18), and plotly (https://plotly.com). All the plots drawn by the website can be downloaded as support vector graphics (.svg) images for subsequent use. Finally, users can also retrieve the results directly from the database via a Python (26) interface, if custom analyses are to be performed.

The code is available on GitHub (https://github.com/metagenlab/zDB), and zDB can be installed as a conda package from the bioconda channel (27).

### Minimal analyses: quality control, orthology inference, and core genome phylogeny

The minimal set of analyses includes quality control with CheckM v1.2.1 (28), the generation of Blast (29) databases, orthology prediction, and phylogeny inference. zDB takes GenBank ﬁles as input and has currently been tested with the output of Prokka (3), PGAP (30), and Bakta (20). As locally assembled genomes may have duplicated accessions or locus tags, zDB ﬁrst checks their uniqueness and automatically generates new identiﬁers if necessary. Amino-acid and nucleotide sequences are then extracted from the GenBank ﬁles and used as input for subsequent analyses (4a). Annotations such as gene names and protein products are also extracted from the GenBank ﬁles. Those annotations are indeed particularly valuable when reference genomes are analyzed together with draft genomes, as the annotations of the genes from a reference genome may hint at the function of their homologs in draft genomes.

Orthology is inferred using Orthoﬁnder v2.5.2 (19). The sequences of orthologous proteins are aligned with MAFFT v7.487 (31), and the alignments are used to infer phylogenetic trees for each orthogroup using FastTree v2.1.8 (32). In addition to gene phylogenies, zDB also generates a species tree with FastTree using the concatenated alignments of the single-copy core orthologs. As some assemblies may be incomplete, the condition that core orthologs must be present in all genomes can be relaxed to allow missing genes. zDB generates Blast databases with both amino-acids and nucleotides sequences for each individual genomes and for the whole data set. This allows users to search for sequences in a speciﬁc genome without the interference of better hits in other genomes, while still making it possible to perform global searches on the whole data set.

### Optional analyses: homology search, COG, KEGG, and Pfam annotations

To complement the core analysis, zDB can perform optional analyses focused on function prediction. Optional analyses all take the proteins of the non-redundant pan-genome as input and include the assignment to the COG, mapping to the KEGG, prediction of Pfam protein domains, and search for homologs in the SwissProt database. COG annotations oﬀer clues regarding protein functions and allow their classiﬁcation in broad functional categories. The assignment to COG (33) clusters is performed by rps-blast (29) searches using the position-speciﬁc score matrices of the NCBI Conserved Domain Database (CDD) (34). KEGG annotations give insights into the metabolic capacities of the analyzed bacteria. The mapping to KEGG orthologs is performed by Kofamscan (35) using the prokaryotic proﬁles of the KEGG database (36). As they can oﬀer functional insights into otherwise unannotated proteins and as domain architecture conservation may be a valuable addition to a gene phylogeny, Pfam protein annotations were also added in the optional analysis. The annotation is performed with the Pfam_scan (37) tool and the Pfam-A database. Finally, zDB can also perform a homology search with blastp (29) against the manually curated entries of the SwissProt (38) database. The reference database used by zDB to perform that analysis is listed in Table 1. Of note, the core analyses can be performed without any reference database.

**TABLE 1:** Reference databases used by zDB

Database	Release	Size*^a^*	Search tool
Swissprot (38)	Release 2021_04	86M	Blastp v2.9.0 (29)
Refseq nr^*b*^ (39)	Release 210	34.9G	Diamond v2.0.13 (40)
KEGG hmm proﬁles (36)	Release 03/2022	1.2G	Kofamscan v1.3.0 (35)
CDD (34)	Release 3.19	4.0G	Rpsblast v2.9.0 (29)
Pfam-A hmm proﬁles (37)	Release 35.0	279M	Pfam_scan v1.6 (37)

^*a*^ Refers to the volume of data to download.

^*b*^ As downloading the non-redundant RefSeq database is prone to failure, it is not automatically downloaded by zDB. A script to download and prepare the databases is installed with zDB but has to be run manually.

To screen for lateral gene transfers using a well-validated method (41), zDB can search the RefSeq database for homologs of proteins from the non-redundant pangenome. The search is performed by Diamond (40) to reduce the duration of the analysis. The proteins of every orthogroup and their best hits (the best four hits of every protein, by default) are then aligned with MAFFT, and the alignment is used by FastTree to infer a phylogenetic tree. As reference genomes downloaded from RefSeq may have been included in zDB input data set, the best hits from genomes already present in the database are ﬁltered out. If the database was populated with genomes of related bacteria, observing that a protein from a distant taxa clusters more closely than the other proteins from the same orthogroup may indeed indicate a lateral gene transfer.

### Benchmarking

Although the analysis pipeline could process more genomes, the visualization platform is designed for data sets ranging from tens to hundreds of genomes. Therefore, we chose a representative data set composed of the NCBI’s 179 reference genomes of the *Enterobacteriaceae* family to benchmark the analysis pipeline. The genomes were downloaded as Genbank ﬁles from the NCBI (Table S1). We ran a ﬁrst benchmark to measure the running time of the diﬀerent optional analyses on the full data set. As the search for RefSeq homologs proved to be prohibitively long (Fig. 4C), it was not included in the subsequent benchmarks. The pipeline was then run on randomly generated subsets of the 179 genomes composed of 10, 20, 40, 90, or all genomes, all with a mean genome size of 3.8 Mbp.

The performances of the pipeline were measured using Nextﬂow with report option. All analyses were run on an Ubuntu 18 server (112 Intel Xeon Platinum 8280 2.7 GHz CPUs, equipped with 377 GB of RAM (random-access memory)), limiting parallelization to 20 simultaneous processes (with Nextﬂow cpus option) and total memory usage to 32 GB (with Nextﬂow memory option), to mimic the computing power of a high-end desktop computer. We also tested the 90 genomes data set on a desktop computer with six cores and 16 GB of RAM memory to have a better idea of the performances of zDB on a computer with more limited resources.

## SUPPLEMENTAL MATERIAL

Fig. S1 msystems.00473-24-s0001.docx The species tree generated by zDB, annotated with the main characteristics of the included genomes.

Table S1 msystems.00473-24-s0002.xlsx All the genomes used for the benchmarking of zDB.

ASM does not own the copyrights to Supplemental Material that may be linked to, or accessed through, an article. The authors have granted ASM a non-exclusive, world-wide license to publish the Supplemental Material files. Please contact the corresponding author directly for reuse.
