# Supplementary material for: zDB: bacterial comparative genomics made easy
Source: mSystems. 2024 Jun 28;9(7):e00473-24. doi: 10.1128/msystems.00473-24 (PMC11264898; doi:10.1128/msystems.00473-24)
Supplement: Fig. S1 — The species tree generated by zDB, annotated with the main characteristics of the included genomes. [file msystems.00473-24-s0001.docx]

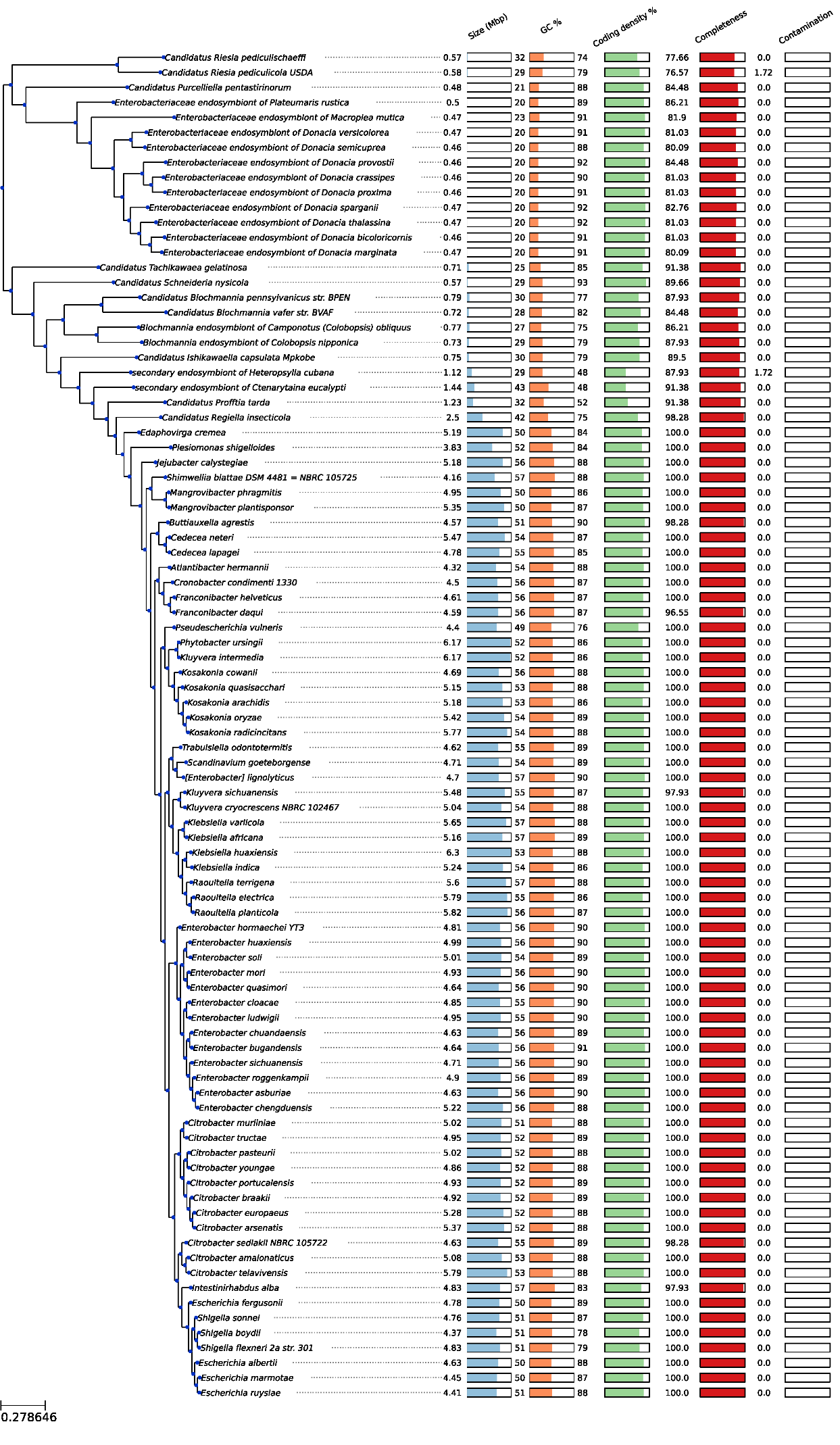


**Supplementary figure S1.** The species tree generated by zDB, annotated with the main characteristics of the included genomes.
